# Supplementary material for: Content-rich biological network constructed by mining PubMed abstracts
Source: BMC Bioinformatics. 2004 Oct 8;5:147. doi: 10.1186/1471-2105-5-147 (PMC528731; doi:10.1186/1471-2105-5-147)
Supplement: Additional File 5 — The original Chilibot query results of the term "long-term potentiation (LTP)" and 22 other terms, limiting the latest references analyzed to the years 1990, 1995, 2000, and 2004. [file 1471-2105-5-147-S5.bz2 › chilibotAdditionalFile5/ltp2004/html/TRKB_NMDA.html]

 


 **TRKB** and **NMDA** 
  
Found 47 abstracts in PubMed,  **30 abstracts were retrieved and analyzed**.  


---

 Search Google  |
 PDF files only 
|  EDU domain only 

---

**Interactive relationship** (e.g. stimulation, inhibition, etc)

- **NMDA**  mediated activation of Akt was completely blocked by MK 801 and partially blocked by the  **TrkB**  receptor inhibitor, K252a, indicating the requirement of  **TrkB**  receptors for maximal activation by  **NMDA** .  Ref: 12373512 Amino Acids, 2002
- Our results demonstrate that the signaling cascade of  **TrkB**  leads to increase in  **NMDA**  receptor activity.  Ref: 14720220 J Neurochem, 2004
- Our findings suggest the importance of interaction between BDNF  **TrkB**  signaling and  **NMDA**  receptors for spatial memory in the hippocampus.  Ref: 12663749 Learn Mem< MedlineTA>Learn Mem, 2004
- Furthermore, recent findings suggest the importance of interaction between BDNF  **TrkB**  signaling and  **NMDA**  receptors for spatial memory.  Ref: 12719654 J Pharmacol Sci, 2003

**Parallel relationship** (e.g. studied together, co-existance, homology, etc.)

- Both  **NMDA**  and  **TrkB**  receptors activate nuclear factor kappaB NF kappaB, a transcription factor known to be involved in protecting neurons against many different kinds of toxic insults.  Ref: 12853306 Ann N Y Acad Sci< MedlineTA>Ann N Y Acad Sci, 2003
- **NMDA**  receptor subunits, trkA and  **trkB** ... were present in spinal cord PSD.  Ref: 11338211 Neuroreport, 2001
- Neither the enhanced nor the reduced  **trkB**  activity influenced MK 801 induced neurotoxic effects in the posterior cingulate cortex of mouse brain, which indicates that increased BDNF production neither protects nor exacerbates neurotoxic effects of  **NMDA**  receptor antagonists.  Ref: 12962903 Neurosci Lett, 2003
- The results indicate that acute exposure to BDNF increases  **NMDA**  single channel open probability via postsynaptic  **trkB**  receptors.  Ref: 11054804 J Neurosci Res, 2000
- Therefore, the PI 3 kinase Akt pathway is co activated by  **NMDA**  and  **TrkB**  receptors.  Ref: 12373512 Amino Acids, 2002
- By converging on survival transcription factors, such as NF kappaB,  **NMDA**  and  **TrkB**  receptors protect neurons.  Ref: 12853306 Ann N Y Acad Sci< MedlineTA>Ann N Y Acad Sci, 2002
- Whereas this cascade may play an important role in the modulation of  **NMDA**  receptors in physiologic conditions, in the context of  **TrkB**  overactivation, it may contribute to neuronal death.  Ref: 14720220 J Neurochem, 2004
- Here, we show that Fyn is a key molecule linking the BDNF receptor  **TrkB**  with  **NMDA**  receptors, which play an important role in spatial memory formation in a radial arm maze.  Ref: 12663749 Learn Mem< MedlineTA>Learn Mem, 2004
